# Evaluation of Hair Density and Hair Diameter in the Adult Thai Population Using Quantitative Trichoscopic Analysis

**DOI:** 10.1155/2020/2476890

**Published:** 2020-02-10

**Authors:** Kanchana Leerunyakul, Poonkiat Suchonwanit

**Affiliations:** Division of Dermatology, Faculty of Medicine, Ramathibodi Hospital, Mahidol University, Bangkok, Thailand

## Abstract

The data of hair density and hair diameter in the Asian population, especially in Thais, are limited. We aimed to evaluate hair density and hair diameter of members of the Thai population at different scalp sites and to determine the effect of sex and aging as well as to compare the results with those in groups of other ethnicities. Healthy Thais whose hair examination findings were normal were evaluated. Two hundred and thirty-nine subjects participated in this study, of whom 79 were male and 160 were female. Hair density and hair diameter were analyzed at four different scalp sites using quantitative trichoscopic analysis. The highest hair density in Thais was observed in the vertex area. Hair densities at four different scalp sites were significantly different from one another; only hair density at the vertex site showed no significant difference from that in the occipital area. In contrast, hair diameter did not show any statistically significant differences for the different sites. We observed decreased mean hair density with increasing age and found statistically significant differences between participants in their 20s and those in their 60s, while hair diameter remained consistent. Comparing our results with a previous study in other ethnicities, the hair densities in Asians are generally lower. In conclusion, hair density in the Thai population varies at different scalp sites. Aging is a factor in declining hair density. Asians have a lower hair density compared to Caucasian and African populations.

## 1. Introduction

Studies determining normal values of hair density and diameter have been conducted in various ethnic groups. Evaluating normative values for scalp hair is beneficial for the diagnosis of hair disorders and monitoring treatment outcomes. Furthermore, the values would be applicable for research proposes. Currently, there are several invasive and noninvasive methods for evaluating hair density and hair diameter.

Skin biopsy is one of the invasive methods used for determining hair parameters in clinical practice and several research studies. However, it is not the preferred choice for hair and scalp examination due to related pain and scars. Therefore, noninvasive techniques such as the wash test, trichoscopy, and phototrichogram are more suitable [1]. Quantitative trichoscopic analysis is a form of videodermoscopy combined with an image analysis system which has the ability to provide hair density and hair diameter. Not only it is an uncomplicated, easily reproducible, and less time-consuming method, it produces accurate results, especially when incorporated with computer-assisted analysis [2–4].

Previous studies using scalp biopsy suggested that hair density and diameter varied at different sites of the scalp [5, 6], and this has been confirmed by several research studies using computer-assisted phototrichograms [7–11]. Another factor reported to affect hair density and hair diameter is aging. Skin biopsies revealed a decrease in hair counts and number of follicular units in subjects of older age [5, 12, 13]. In addition, phototrichogram studies confirmed that hair density and hair diameter reduced with chronological aging [7, 14–17]. While hair parameters were slightly different between genders, none of these differences were statistically significant [5, 13, 17–19].

Ethnic differences also impact variation in hair density and hair diameter. Previous studies using both invasive and noninvasive methods have observed interracial differences [5, 10, 13, 17–19]. A recent study by Birnbaum et al. using quantitative trichoscopic analysis supported this variation, by performing comparisons among Caucasian, African, and Hispanic populations [10]. However, data on an Asian population using the same evaluation method are still lacking. Our study aimed to evaluate hair density and hair diameter at different scalp sites in the normal Thai population and to determine the effect of sex and aging on both values, as well as to compare the hair density of this Asian population with those of other ethnicities.

## 2. Materials and Methods

This study was approved by the Mahidol University Institutional Review Board for Ethics in Human Research (lD 02-61-56), and written consent was received from each participant before enrollment. Subjects were recruited from the outpatient clinic at Ramathibodi Hospital in Bangkok between April 2017 and February 2018. Inclusion criteria were healthy volunteers aged 18 years or more. Exclusion criteria included self-reported hair loss within 6 months prior to the study, having underlying systemic conditions, having a nonAsian ancestor, having a history of using medication or hair products that affect the hair growth cycle, a positive hair pull test, abnormal hair or scalp observed on physical examination, and those with abnormalities detected on trichoscopy. The sample size was estimated based on data from a previous normative study [11], to achieve a power of 80% and a confidence level of 95%; the calculated minimum number of participants was 218.

The subject's hair was parted in the midline. Three sites on the scalp were then measured at 12, 24, and 30 cm from the glabella, similar to in Birnbaum et al.'s study, which were identified as the frontal, vertex, and occipital area [10]. The measurement point of the temporoparietal area was located 6 cm from the external ear canal on the side of the subject's scalp. Trichoscopic pictures were taken by the Folliscope^®^ (LeadM Corporation, Seoul, Korea) at these four scalp sites. The hair density was evaluated at 50-fold magnification, while the hair diameter was evaluated from 100-fold magnification images. All hair parameters were determined by manual measurements.

Statistical analysis was performed using the SPSS statistical software, version 18.0 (SPSS Inc., Chicago, IL). The differences in hair density and hair diameter at different scalp sites, among age groups, and between male and female participants as well as a comparison of hair density with densities found in those of different ethnic groups in the previous study by Birnbaum et al. [10] were evaluated using analysis of variance. Post hoc pairwise comparison was conducted using Tukey's Honestly Significant Difference test after determining differences in each series. Statistical significance was determined as *P* < 0.05.

## 3. Results

Two hundred and thirty-nine subjects were recruited to participate in this study, 79 men and 160 women with an average age of 37.9 years. The mean hair densities at the frontal, vertex, temporoparietal, and occipital areas were 154.3 ± 12.8, 162.9 ± 15.7, 133.7 ± 14.6, and 160.2 ± 15.3 cm^2^, respectively. Pairwise comparisons between different scalp areas showed that almost all pairs of scalp sites had statistically significant differences (*P* < 0.05), except the comparison between the vertex and occipital areas. Regarding hair diameter, none of the pairs from two different areas was statistically significantly different after being analyzed with pairwise comparisons (Table 1).

When focusing separately on each sex, hair density in both male and female participants showed comparable patterns as being greatest in the vertex and lowest in the temporoparietal area (Table 2). Neither hair density nor hair diameter showed any statistically significant differences between male and female participants.

**Figure 1:**
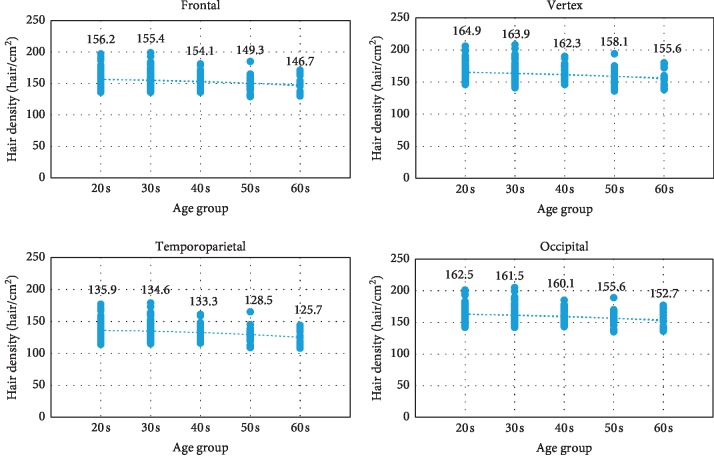
Comparisons of hair density in different age groups at different scalp areas.

Participants were stratified into five subgroups according to their age: 20s (*n* = 78), 30s (*n* = 71), 40s (*n* = 45), 50s (*n* = 27), and 60s (*n* = 18). The mean ages of participants in these groups were 25.78, 34.1, 43.8, 53.5, and 67.4 years, respectively. Hair density tended to decrease with increasing age (Figure 1). Pairwise comparisons revealed that only those in their 20s and 60s had differences in hair density at all scalp areas (Table 3). In contrast, no statistical difference in hair diameter was observed among different age groups.

Further analysis was performed to compare hair density in our study with a previous study that used the same measurement method (Table 4) [10]. The hair densities in the Asian group were relatively lower than that of those in other ethnicities at all scalp areas, except for lower hair densities in the vertex and occipital areas in the African population. Almost all comparisons showed statistically significant differences (*P* < 0.01), except the comparison of hair density in the frontal area between the Asian and African populations (*P*=0.39). Caucasian and African subjects displayed the highest hair density in the frontal area, while the Hispanic and Asian groups shared the highest index in the vertex area.

## 4. Discussion

Our study provides the average hair density and hair diameter of Asian participants in different scalp areas and evaluates factors that potentially have an influence on hair parameters, including sex, aging, and ethnicity. The highest hair density in Thais was observed in the vertex area, followed by the occipital, frontal, and temporoparietal areas. Hair densities at all scalp sites showed statistically significant differences, but hair densities in the vertex and occipital areas were not different to each other. The largest hair diameter was found in the frontal area, but this was not statistically significantly different to the hair at the other areas. These findings were consistent in both males and females. We also demonstrated findings consistent with previous studies that age affects hair density reduction and that there is a diversity in hair density among different ethnicities.

Our results also confirmed the findings from previous studies that hair density varies at different sites on the scalp. A study conducted in Taiwan using 4 mm scalp biopsies found that the total number of hairs was different in each scalp area, but this difference was not significant [5]. In that study, the density was the greatest in the frontal area and lowest in the temporal area. Another similar research study from Brazil included specimens from the vertex area and displayed similar findings with lower values, and their greatest parameters were observed at the vertex area [6]. Previous studies using automated phototrichograms revealed that the highest hair density was in the midscalp area, while the parietal area presented the lowest hair density [11, 15]. While our research acknowledged the differences among scalp sites, the highest density was seen in the vertex area and the lowest density in the temporoparietal area.

The different hair densities in different scalp areas could be explained by the fact that the hair follicle develops during the embryonic stage through multiple interactions between epithelial and mesenchymal cells [20]. During the initial phase of hair follicular morphogenesis, many signals, including the Wnt pathway, transduce from the mesenchymal cells to form a placode which then leads to development of the hair germ. We hypothesize that the number of mesenchymal cells transcending the signals may be related to the variations in hair follicles in different scalp sites. In contrast, the hair diameter remained consistent, representing a similar follicular size at all areas of scalp, and remained so among different individuals of the same ethnicity. There has been speculation that human hair diameter is dictated by specific genes that vary among different ethnicities. The hair diameter in Asians is strongly determined by ectodysplasin-A receptor gene variance. Those with CC genotypes would have a greater hair diameter with larger cross-sectional areas than those with TC and TT genotypes [21–23].

We also found that gender difference does not affect hair density and hair diameter. Aslani et al. reported nonsignificant differences in hair counts between males and females in a Caucasian population [13]. Two similar studies in Asians also observed no differences [5, 19]. Our results showed similar patterns in both hair density and hair diameter in the two sexes. The highest hair density was seen in the frontal area, and the lowest hair density was in the temporoparietal area in both genders, whereas hair diameter in both genders was consistent at all sites. This leads to the hypothesis that hormones are not involved in development and functions of hair follicles or keratinization of hair shafts in individuals without androgenetic alopecia (AGA). We also believe that in normal people, both sexes share the same genetic factors for proliferation of hair. This is unlike the case in patients suffering from male AGA or female pattern hair loss (FPHL), in whom hair density and hair diameter decrease differently. Multiple studies suggest that male AGA is caused by numerous polymorphisms associated with androgen-related genes, while FPHL may have a different etiology and may not be linked to a hormone-driven mechanism [24].

It is well known that the frequency of hair loss increases with aging. In the absence of hair loss, several previous studies found that age differences affect hair density and hair diameter. Previous studies in Asians using computer-assisted phototrichograms reported a decline in hair density in normal Japanese females over 40 years of age [14]. A similar observation was made in Korean studies [8]. In comparing hair diameter among different age groups, Japanese females in their 20s showed a different hair diameter from that in their 40s. However, while Kim et al. observed that reduction in hair diameter started from the 40s onward, this was not statistically significant [8]. Meanwhile, Trotter and Atkinson et al. observed that in the white population hair diameter reached its peak during early adulthood and began to drop with increasing age [25, 26]. Our study demonstrated decreased hair density with increasing age, but the level of reduction was not large enough to detect statistical significance until participants reached their 60s. Moreover, we found contrasting results in terms of hair diameter compared to other previous studies. We observed no statistical difference in hair diameter in the different age groups. Our results further suggested that aging causes hair follicular stem cells to undergo senescence and to lose their self-renewal capacity without diminishing cross-sectional area. These findings correspond to senescent alopecia, which presents with diffused hair loss in those at least 50 years of age [12, 27–29]. The aged hair follicles failed to reenter the hair growth phase due to a lengthened telogen phase and shortened anagen phase without follicular miniaturization. Specimens from senescent scalp showed lower expression of genes associated with transcription and growth factors and downregulation of signals linked to the anagen phase [30]. The alteration of signals regulating the microenvironment of the stem-cell niche and DNA damage from repetitive hair cycling are also involved in hair reduction with aging [28]. In general, senescent alopecia can coexist with AGA or FPHL, as it presents only in the senior population. However, the constant measurement of hair diameter observed in our research clearly indicated that we excluded people with AGA, which shows a diversity of hair diameters.

With regard to ethnic variances, our findings support the diversity of hair density and hair diameter among different ethnic groups. Comparing our results to those of a previous study [10], the hair densities in Asians differ from those in Caucasian, African, and Hispanic populations. We believe that racial factors and genetic backgrounds are the main contributors to the follicular reservoir and hair counts, as well as the distributions of hairs. However, the fact that there were lower numbers of subjects in the Caucasian and African groups in the previous study should also be taken into consideration in interpreting these pattern variations. Moreover, hair diameter was not included in the previous study in the other ethnicities, so we could not perform a comparison of hair diameter among different ethnicities. Therefore, a study with a large sample size should be performed in future to evaluate the pattern of hair density and hair diameter among different locations in persons of various ethnicities.

Our study was conducted at a single tertiary care center and therefore may not represent the general Thai population. A further nationwide cross-sectional study with stratification could be performed to eliminate this limitation.

## 5. Conclusion

The findings of the present study agreed with those of previous studies that hair density varied at different sites of the scalp and among persons of different ethnic origins. Hair density progressively decreases with age, statistically significantly so in those in their 60s. However, the hair diameter at different sites remained unchanged and was not affected by sex or aging. Taken together, hair density and hair diameter are distinctive in the Asian population. It is important to take this reference index into account before making a diagnosis of hair loss in Asian persons and for setting an accurate treatment goal. Our findings will also help researchers establish study endpoints.

## Figures and Tables

**Table 1:** Comparisons of hair density and hair diameter among different scalp sites.

	Scalp area				
	Frontal	Vertex	Temporoparietal	Occipital	*P* value^†^	

Hair density (hairs/cm^2^)	154.3 ± 12.8	162.9 ± 15.7	133.7 ± 14.6	160.2 ± 15.3	<0.001^*∗*^	
Hair diameter (*μ*m)	81.1 ± 6.9	80.8 ± 5.9	80.1 ± 4.5	80.3 ± 5.1	0.066	

	Pairwise comparison					
	Frontal vs. vertex	Frontal vs. occipital	Frontal vs. temporoparietal	Vertex vs. occipital	Vertex vs. temporoparietal	Occipital vs. temporoparietal

Hair density (hairs/cm^2^)	*P* < 0.01^*∗*^	*P* < 0.01^*∗*^	*P* < 0.01^*∗*^	*P*=0.17	*P* < 0.01^*∗*^	*P* < 0.01^*∗*^
Hair diameter (*μ*m)	*P*=0.81	*P*=0.07	*P*=0.19	*P*=0.41	*P*=0.69	*P*=0.97

Data are mean ± SD. ^*∗*^Statistically significant difference. ^†^Analysis was performed using ANOVA.

**Table 2:** Comparisons of hair density and hair diameter between males and females.

Scalp area	Hair density (hairs/cm^2^)			Hair diameter (*μ*m)		
	Male	Female	*P* value	Male	Female	*P* value
Frontal	155.2 ± 15.7	153.9 ± 13.5	0.46	81.3 ± 4.4	81.2 ± 5.1	0.76
Vertex	163.5 ± 14.1	162.6 ± 12.9	0.6	81.1 ± 6.5	80.7 ± 6.3	0.56
Temporoparietal	134.6 ± 16.7	133.2 ± 14.2	0.4	80.2 ± 5.8	80.3 ± 4.7	0.87
Occipital	161.3 ± 15.5	160.1 ± 13.6	0.49	80.1 ± 3.6	80.1 ± 6.9	0.31

Data are mean ± SD.

**Table 3:** Comparisons of hair density and hair diameter with aging among different scalp sites.

Age group	Frontal	Vertex	Temporoparietal	Occipital
Hair density (hairs/cm^2^)				
20s	156.2 ± 12.8	164.9 ± 12.7	135.9 ± 12.6	162.5 ± 12.7
30s	155.4 ± 13.4	163.9 ± 13.6	134.6 ± 13.8	161.5 ± 13.3
40s	154.1 ± 10.2	162.3 ± 10.5	133.3 ± 10.2	160.1 ± 10.3
50s	149.3 ± 11.3	158.1 ± 11.8	128.5 ± 11.3	155.6 ± 11.4
60s	146.7 ± 14.5^*∗*^	155.6 ± 14.5^*∗*^	125.7 ± 13.7^*∗*^	152.7 ± 14.5^*∗*^

Hair diameter (*μ*m)				
20s	81.4 ± 4.4	81.1 ± 4.5	80.1 ± 4.6	80.6 ± 4.9
30s	80.9 ± 5.4	80.5 ± 5.6	78.2 ± 5.2	80.3 ± 5.4
40s	80.2 ± 5.2	80.1 ± 5.3	79.6 ± 4.5	79.1 ± 5.6
50s	80.7 ± 5.2	80.4 ± 5.2	79.9 ± 5.1	79.8 ± 5.5
60s	82.4 ± 4.6	82.2 ± 4.8	81.2 ± 5.3	81.2 ± 4.4

Data are mean ± SD. ^*∗*^*P* < 0.05, statistically significant difference when compared to 20s.

**Table 4:** Comparisons of hair density and hair diameter with aging among different scalp sites.

Scalp area	Ethnicity				Pairwise comparison		
	Asian (present study)	Caucasian	African	Hispanic	Asian vs. Caucasian	Asian vs. African	Asian vs. Hispanic
Frontal	154.3 ± 12.8	230 ± 33	160 ± 27	174 ± 32	*P* < 0.01^*∗*^	*P*=0.39	*P* < 0.01^*∗*^
Vertex	162.9 ± 15.7	226 ± 20	149 ± 23	178 ± 33	*P* < 0.01^*∗*^	*P* < 0.01^*∗*^	*P* < 0.01^*∗*^
Occipital	160.2 ± 15.3	214 ± 28	148 ± 25	169 ± 31	*P* < 0.01^*∗*^	*P* < 0.01^*∗*^	*P* < 0.01^*∗*^

Data are mean ± SD. ^*∗*^Statistically significant difference.

## Data Availability

The data used to support the findings of this study are available from the corresponding author upon request.
